# Response to dabrafenib and trametinib combined with pembrolizumab in an elderly patient with lung adenocarcinoma of unknown primary harboring BRAF V600E mutation and high PD-L1 expression: a case report

**DOI:** 10.3389/fimmu.2025.1666461

**Published:** 2025-11-17

**Authors:** Nan Li, Jing Hu, Guangjin Bao, Zhigang Cai

**Affiliations:** 1 Department of Cardiothoracic Surgery, Naval Medical Center, Naval Medical University, Shanghai, China; 2 Department of Radiology, Naval Medical Center, Naval Medical University, Shanghai, China

**Keywords:** BRAF V600E mutation, non-small cell lung cancer, immunotherapy, targeted therapy, dabrafenib, trametinib, case report

## Abstract

BRAF V600E mutation defines a rare but targetable subset of NSCLC. We report a 70-year-old non-smoking woman with unknown primary lung adenocarcinoma presenting with multistation mediastinal lymph-node metastases and massive malignant pleural and pericardial effusions. Molecular profiling showed BRAF V600E mutation and high PD-L1 expression(TPS 90%, CPS 95). The patient received combined dabrafenib, trametinib, and pembrolizumab with close safety monitoring, achieving rapid tumor control and complete remission by six months with manageable toxicity. This case suggests that early integration of PD-1 blockade with BRAF/MEK inhibition treatment may benefit selected patients and underscores the value of comprehensive molecular and immunohistochemical assessment to guide individualized therapy.

## 1 Introduction

Lung adenocarcinoma is the most prevalent subtype of non-small cell lung cancer (NSCLC) and remains a main cause of cancer-related mortality worldwide (1). Molecular profiling and immunohistochemical analyses have dramatically transformed the therapeutic landscape of NSCLC, enabling the use of personalized therapies targeting specific oncogenic drivers (2). Among these, the v-raf murine sarcoma viral oncogene homolog B (BRAF) mutation represents a relatively rare but clinically actionable alteration, present in approximately 2-4% of NSCLC cases (3). BRAF and MEK inhibitors—represented here by dabrafenib and trametinib—were small-molecule agents that blocked the MAPK (RAF–MEK–ERK) pathway and were commonly used in combination in BRAF V600E–mutant NSCLC (4). Simultaneously, high expression of programmed death-ligand 1 (PD-L1) predicts a favorable response to immunotherapy using PD-1/PD-L1 inhibitors (5). Related studies indicate promise for combining targeted therapy with immunotherapy to achieve durable responses, but prospective and real-world data in BRAFV600E-mutant NSCLC remain limited, especially for concurrent first-line initiation (6). Here, we report a rare case of an elderly non-smoking female patient presenting with lung adenocarcinoma of unknown primary with extensive lymph node metastases and massive malignant pleural and pericardial effusions, harboring a BRAF V600E mutation and high PD-L1 expression, who exhibited a remarkable response to combined dabrafenib, trametinib and pembrolizumab therapy. This case report highlights the potential value of early integration of PD-1 blockade with BRAF/MEK inhibition therapy in selected, high tumor burden cases.

## 2 Case report

**Figure 1:**
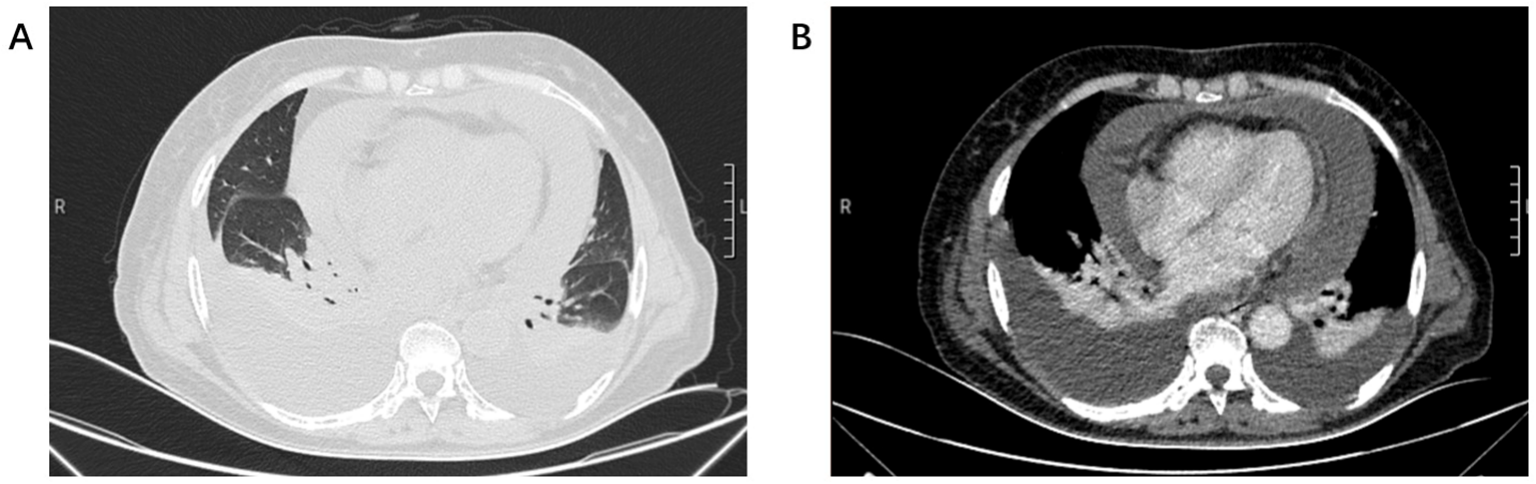
**(A)** Chest CT lung window showed bilateral lung compression and pericardial enlargement. **(B)** Chest CT mediastinal window showed bilateral pleural effusion and pericardial effusion.

A 70-year-old non-smoking female presented to our hospital with complaints of progressive chest tightness and dyspnea. Physical examination revealed diminished breath sounds bilaterally and distant heart sounds. Chest computed tomography (CT) demonstrated massive bilateral pleural and pericardial effusions, bilateral lung compression with atelectasis, and multiple enlarged lymph nodes in the mediastinum, hilum, and cervical regions (**Figure 1**). To alleviate her severe dyspnea, thoracentesis and pericardiocentesis were performed, yielding serosanguinous fluid. Cytological analysis of pleural and pericardial effusions showed a few atypical cells suspicious for malignancy. Tumor marker analysis of the effusions revealed CEA at 0.39 ng/ml(ref.<5), CA-125 >1000 U/ml(ref.<35), cytokeratin 19 fragment at 22.9 µg/L(ref.<3.3), and squamous cell carcinoma antigen at 3.6 ng/ml(ref.<1.5). Serum tumor markers included CA-199 at 44.87 U/ml(ref.<37) and CA-125 at 539.9 U/ml(ref.<35).

**Figure 2:**
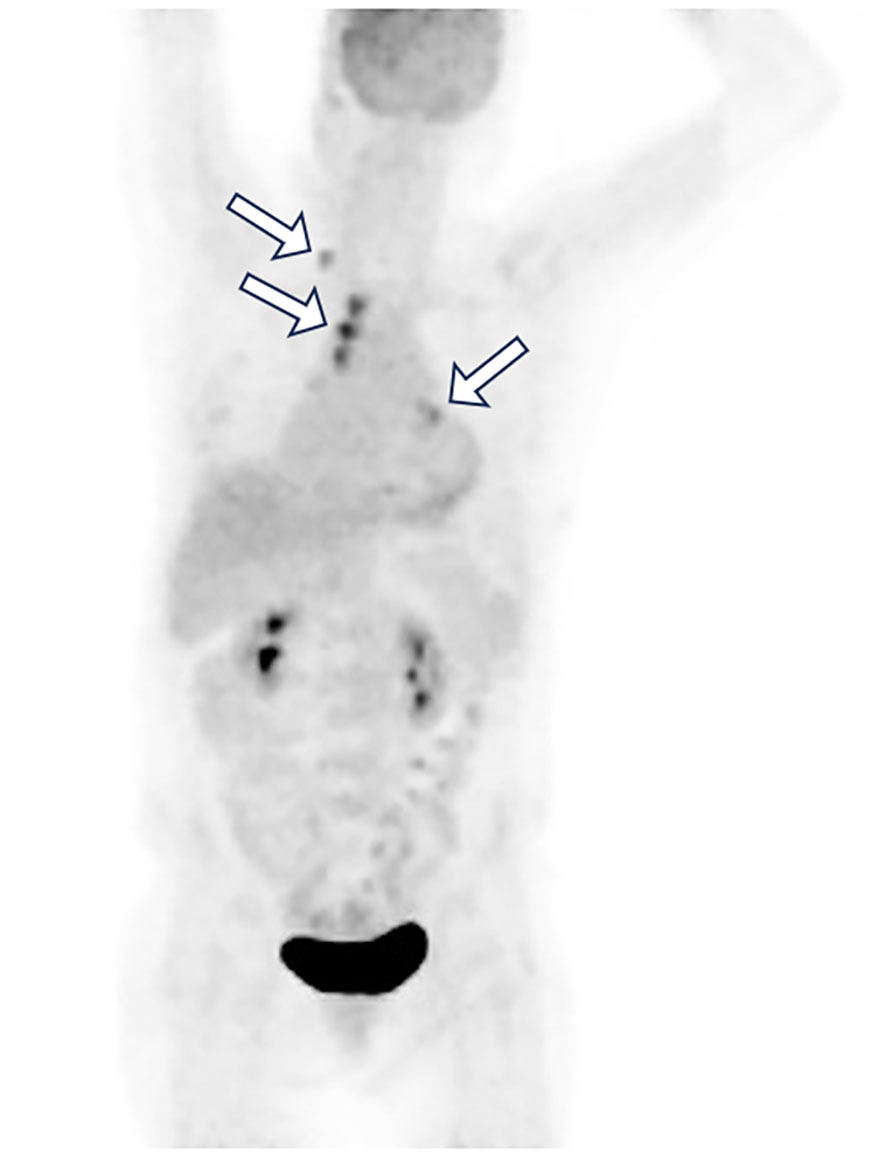
Whole-body PET-CT image revealed multiple hypermetabolic lymph nodes (white arrow) in the right supraclavicular, bilateral hilar, and mediastinal regions, but no definitive primary mass.

**Figure 3:**
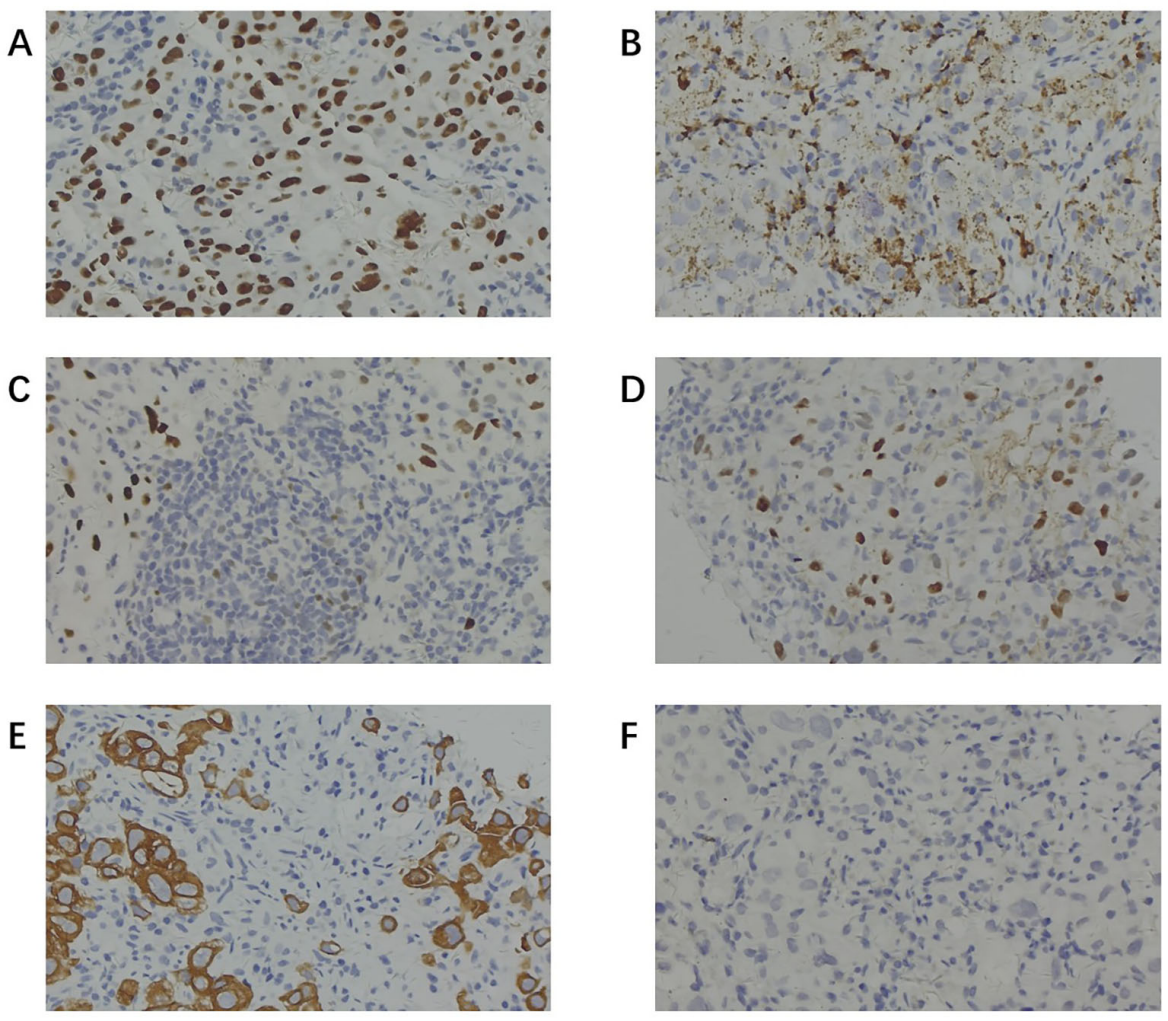
Immunohistochemical staining of lymph node biopsy specimens. **(A)** Positive staining for TTF-1. **(B)** Positive staining for Napsin A. **(C)** Partially positive staining for P63. **(D)** Partially positive staining for P40. **(E)** Positive staining for CK-7. **(F)** Negative staining for CDX-2. (TTF-1 and Napsin A positivity (with CK7) supported pulmonary adenocarcinoma; P40 and CK5/6 negativity argued against squamous differentiation; CDX2 and CK20 negativity helped exclude a lower gastrointestinal primary; and PAX8 negativity argued against renal/gynecologic/thyroid origins).

To further investigate the primary lesion and potential metastases, a positron emission tomography–computed tomography (PET-CT) scan was performed, revealing multiple hypermetabolic lymph nodes in the right supraclavicular, bilateral hilar, and mediastinal regions, but no definitive primary mass (**Figure 2**). No neoplasms were found in the trachea or bronchi by fiberoptic bronchoscopy, and no abnormal cells were found in the bronchoalveolar lavage fluid. An ultrasound-guided fine-needle aspiration biopsy of lymph node was performed. Histopathology showed tumor cells positive for TTF-1, Napsin A, CK7, and CAM5.2, partially positive for P63 and P40, negative for PAX8, CDX2, GATA3, SATB2, Arg1, and INSM1 (**Figure 3**). These findings supported a diagnosis of metastatic lung adenocarcinoma with undetectable primary site.

Genomic testing of the lymph node biopsy identified a BRAF V600E missense mutation in exon 15 (abundance: 6.51%), BRCA1 exon 21 missense mutation (abundance: 47.77%), IDH1 exon 4 missense mutation (abundance: 6.19%), SDHA exon 6 frameshift mutation (abundance: 2.73%), and a TP53 exon 9 splice site mutation (abundance: 8.19%). PD-L1 expression analysis demonstrated a tumor proportion score (TPS) of 90% and a combined positive score (CPS) of 95.

Based on clinical and molecular findings, the patient was staged as TxN3M1a, corresponding to stage IVA disease, and deemed inoperable. Given the BRAFV600E mutation and high PD-L1 expression, and with the goals of rapid tumor control and durable response, we initiated dabrafenib (150 mg twice daily), trametinib (2 mg once daily), and pembrolizumab (200 mg every three weeks) concurrently. Predefined safeguards were implemented, including close laboratory and symptom monitoring, prompt management of pyrexia, rash, hepatitis, and pneumonitis, and temporary dose interruptions or reductions as needed.

**Figure 4:**
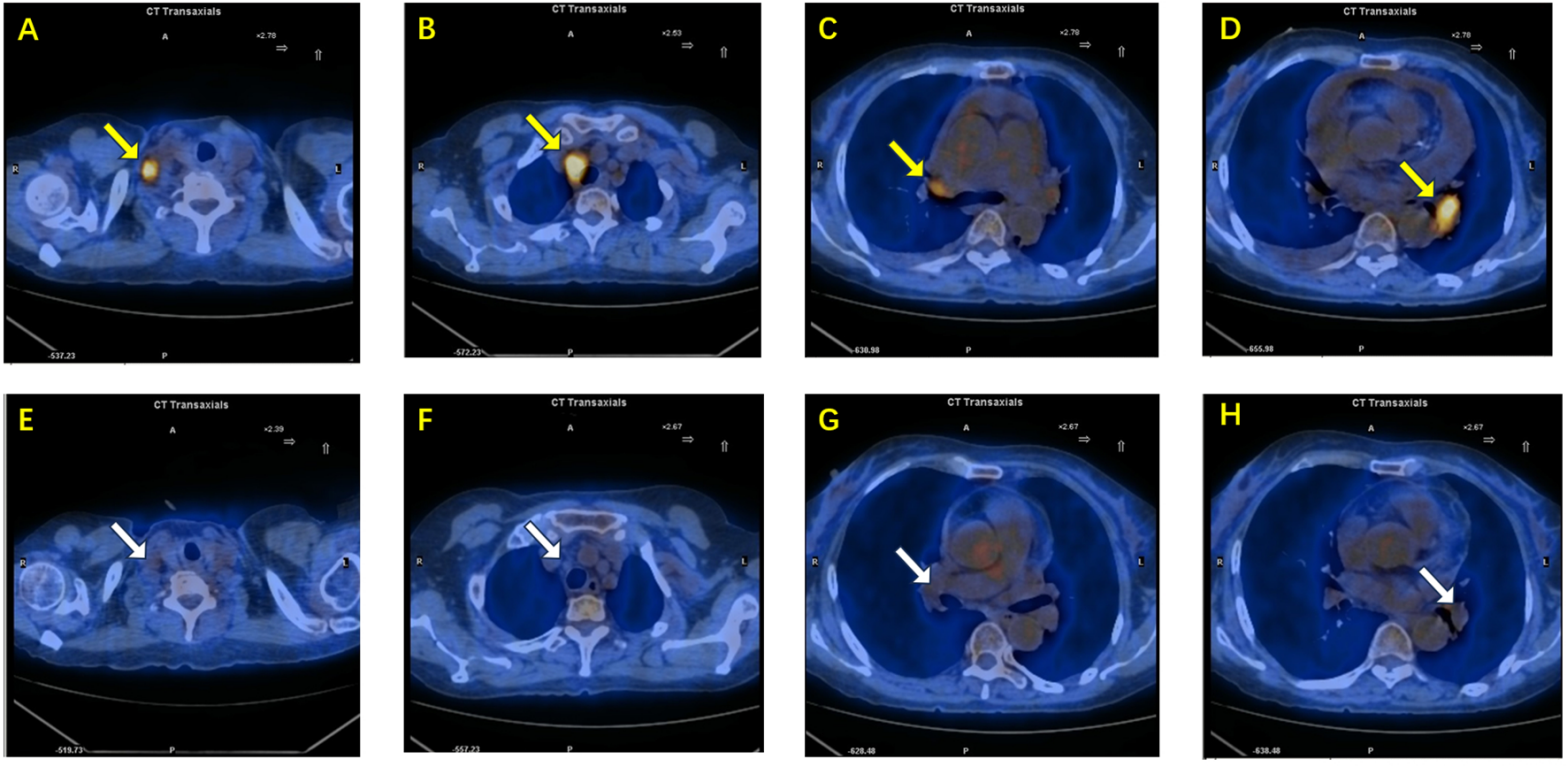
**(A–D)** PET-CT images before treatment demonstrate enlarged lymph nodes with high metabolic activity(yellow arrow): **(A)** right supraclavicular lymph node, **(B)** right paratracheal lymph node, **(C)** right hilar lymph node, and **(D)** left hilar lymph node. **(E–H)** Corresponding PET-CT images after combined targeted and immunotherapy show significant reduction in lymph node size and decreased metabolic activity(white arrow): **(E)** right supraclavicular lymph node, **(F)** right paratracheal lymph node, **(G)** right hilar lymph node, and **(H)** left hilar lymph node.

After four months of continuous therapy, repeat PET-CT imaging demonstrated significant reduction in size and metabolic activity of the previously enlarged lymph nodes (**Figure 4**). The patient reported substantial symptomatic improvement, and no new metastatic lesions were observed. At 6 months, imaging and laboratory (tumor marker) follow-up revealed no signs of tumor relapse. The patient tolerated the treatment well, experiencing only mild liver enzyme elevations.

## 3 Discussion

Cancer of unknown primary (CUP) remains a perplexing and challenging entity in oncology, accounting for approximately 3–5% of all cancer diagnoses worldwide (7). Defined by the presence of metastatic disease without a clinically or radiologically detectable primary site despite extensive investigation, CUP often presents with rapid progression, early dissemination, and poor prognosis (8). CUP cases are broadly categorized into two prognostic subgroups: favorable and unfavorable (9). The favorable subset, constituting about 15–20% of cases, includes patients with specific clinicopathological features who often benefit from treatment strategies tailored to presumed primary tumors. Examples include isolated axillary lymph node metastases in female patients, which are managed similarly to breast cancer, and peritoneal carcinomatosis of serous papillary adenocarcinoma type in females, typically treated following ovarian cancer protocols. Patients within this group frequently achieve significantly improved survival outcomes, in some cases approaching those of corresponding primary cancers. In contrast, the unfavorable subset comprises the majority (around 80–85%) of CUP patients. These tumors are characterized by poor differentiation, rapid progression, unpredictable metastatic spread, and resistance to conventional chemotherapy. Median overall survival in this subgroup remains dismal, often limited to a few months (7). From a biological perspective, two primary hypotheses have been proposed to explain the origin of CUP (10, 11). The first suggests that CUP represents a variant of known primary tumors where the primary lesion is either too small to detect or has regressed. Supporting this view, autopsy studies have revealed occult primaries in a significant proportion of CUP cases. The second hypothesis posits that CUP constitutes a distinct biological entity, characterized by early systemic dissemination and the absence or regression of the primary tumor. This “true CUP” hypothesis suggests shared molecular and clinical features that transcend tissue of origin.

A distinct subset of CUP cases exhibits molecular and immunophenotypic features consistent with a pulmonary origin, termed lung cancer of unknown primary. This group is characterized by the expression of lung lineage markers such as TTF-1 and Napsin A, which are highly sensitive and specific for lung adenocarcinoma (12). Molecular profiling has revolutionized the characterization of lung cancer, particularly by enabling the identification of rare oncogenic drivers that occur in ≤5% of non-small cell lung cancers (13). Advances in next-generation sequencing (NGS) have facilitated comprehensive analyses, uncovering diverse alterations such as rare mutations, gene fusions, and copy number amplifications in genes like MET, ERBB2, and BRAF (14). This integrative molecular approach not only refines tumor classification but also guides the development of targeted therapies, ultimately expanding treatment options for patients with rare molecular lung cancer subtypes (15).

The discovery of activating BRAF mutations, particularly the V600E variant, marked a significant milestone in oncology by identifying critical drivers of tumorigenesis in NSCLC, where they represent a rare but important oncogenic alteration found in approximately 2–4% of cases (16). These mutations induce constitutive activation of the rat sarcoma viral oncogene homolog (RAS)/mitogen-activated protein kinase (MAPK) pathway, promoting uncontrolled cellular proliferation and survival (17). Mechanistically, BRAF belongs to the rapidly accelerated fibrosarcoma (RAF) family of serine/threonine kinases and functions within the receptor tyrosine kinase (RTK)-RAS-RAF-MEK- extracellular signal-regulated kinase (ERK) signaling cascade (18). BRAF V600 mutants possess a unique ability to signal as active monomers, a feature that underlies their selective inhibition by RAF inhibitors (19). The introduction of selective BRAF inhibitors such as vemurafenib, dabrafenib, and encorafenib transformed the therapeutic landscape for BRAF V600-mutant cancer, significantly improving response rates and survival outcomes (20). Nonetheless, resistance to BRAF inhibition almost universally emerges, most commonly through reactivation of upstream signaling components. In particular, upregulation or activation of RTKs, such as epidermal growth factor receptor (EGFR), restores MAPK pathway activity by promoting RAS activation and subsequent RAF dimerization, thereby effectively bypassing BRAF inhibition (21). The introduction of combined BRAF and MEK inhibition (e.g., dabrafenib plus trametinib; encorafenib plus binimetinib) has significantly advanced treatment for BRAF V600E-mutant NSCLC (22). This dual blockade not only improves response rates and disease control compared to monotherapy but also reduces certain toxicities linked to paradoxical MAPK activation. Clinical trial data have demonstrated objective response rates exceeding 60% in pretreated and treatment-naïve patients, with meaningful improvements in progression-free and overall survival (23).

Despite these successes, resistance to BRAF-targeted therapies remains a major clinical challenge (24). Mechanisms include both BRAF-dependent alterations (such as secondary mutations, splice variants, and gene amplification) and BRAF-independent pathways including activation of upstream RAS or downstream MEK mutations, and bypass signaling through phosphatidylinositol 3-kinase (PI3K)/protein kinase B (AKT) or MET amplification (25).

Beyond targeted combinations, recent investigations are exploring strategies integrating RAF inhibitors with immunotherapies. Using a syngeneic mouse model of BRAFV600E melanoma, the combination of dabrafenib, trametinib, and adoptive cell transfer (ACT) led to complete tumor regression, superior to any single or dual combination treatment (26). This triple approach enhanced T cell infiltration, increased tumor antigen and major histocompatibility complex (MHC) expression, and maintained T cell effector functions *in vivo*, despite concerns that MEK inhibition might impair immune responses. Additionally, the combination altered the tumor microenvironment by reducing immunosuppressive cell populations, including tumor-associated macrophages and regulatory T cells, which are typically increased following BRAF inhibitor therapy alone. Gene expression analyses further revealed upregulation of immune-related genes and chemokines, supporting a more immunogenic tumor milieu. In a phase 1 study, the feasibility and efficacy of combining BRAF and MEK inhibitors (dabrafenib and trametinib) with the anti–PD-1 antibody pembrolizumab were evaluated in 15 patients with advanced BRAFV600-mutant melanoma who received the triple combination therapy (27). The study demonstrated an encouraging objective response rate of 73%, with 40% of patients maintaining ongoing responses at a median follow-up of over two years. Notably, the triple therapy enhanced intratumoral immune activity, as shown by increased CD8+ T cell infiltration and elevated expression of interferon-related genes and MHC molecules in post-treatment biopsies. These findings indicate that targeted therapy can sensitize tumors to immunotherapy by fostering a more inflamed and antigen-rich tumor microenvironment, providing a clear mechanistic basis for introducing PD-1 blockade therapy early alongside BRAF/MEK inhibition.

The COMBI-i study assessed the safety and early efficacy of combining spartalizumab with dabrafenib and trametinib in advanced BRAF-mutant melanoma (28). The triplet regimen showed promising response rates and manageable toxicity profiles, which were similar to the profiles observed with targeted therapies alone. Biomarker analyses revealed that patients with high T cell-inflamed gene expression signatures and low immunosuppressive tumor microenvironment (TME) scores at baseline were more likely to benefit. Conversely, early disease progression was linked to low tumor mutational burden and a strongly immunosuppressive baseline profile. Accordingly, careful patient selection supported by comprehensive genomic and immune profiling is essential to identify candidates most likely to benefit from the triplet therapy and to time its use appropriately. Beyond PD-L1, co-mutation set has yet to be prospectively validated to select candidates for concurrent BRAF–MEK inhibition with PD-1 blockade therapy in NSCLC. In our case, the co-mutations (BRCA1, IDH1, SDHA, TP53) lacked evidence to guide treatment, and predictive panels for selecting patients still require further study. If the disease recurs, we will obtain a repeat tissue biopsy and perform comprehensive genomic profiling to identify potential targetable drivers or resistance mechanisms and guide subsequent therapy.

In clinically urgent, symptomatic cases with high tumor burden, such as large-volume malignant pleural effusions with lung compression and a pericardial effusion causing tamponade, particularly when PD-L1 expression is high, starting all three agents together may be clinically justified to achieve rapid tumor control while pursuing durability, so long as it is used judiciously with close safety monitoring. By contrast, in lower-burden disease with non-urgent symptoms or limited treatment tolerance, a sequential approach remains reasonable. Cheng et al. (29) reported two cases of unresectable stage III NSCLC harboring BRAF V600E mutations successfully treated with a combination of dabrafenib, trametinib, and a PD-1 inhibitor as induction therapy. Both patients demonstrated significant tumor regression, which allowed for subsequent surgical resection that was initially considered infeasible. Nevertheless, real-world evidence for starting all three agents together in the first line in BRAFV600E-mutant NSCLC remains limited, and our case contributes practice-based support by demonstrating feasibility and meaningful benefit in an elderly, high tumor burden patient.

## 4 Conclusion

We reported an elderly, non-smoking woman with unknown primary lung adenocarcinoma harboring a BRAF V600E mutation and high PD-L1 expression, who presented with high tumor burden including multistation mediastinal lymph-node metastases and massive pleural and pericardial effusions. Combined treatment with dabrafenib, trametinib, and pembrolizumab produced rapid tumor control and complete remission by six months with manageable toxicity. This experience suggests that, in carefully selected patients with BRAF V600E–mutant, high-PD-L1 expression, early integration of PD-1 blockade with BRAF/MEK inhibitor treatment may provide significant clinical benefit. At the same time, the triplet therapy is not a routine standard in NSCLC and should be used with caution, with appropriate patient selection, close safety monitoring. Prospective studies and larger real-world series are needed to confirm benefit and refine the criteria for patient selection.

## Data Availability

The raw data supporting the conclusions of this article will be made available by the authors, without undue reservation.

## References

[1] Sung H Ferlay J Siegel RL Laversanne M Soerjomataram I Jemal A . Global cancer statistics 2020: GLOBOCAN estimates of incidence and mortality worldwide for 36 cancers in 185 countries. CA Cancer J Clin. (2021) 71:209–49. doi: 10.3322/caac.21660, PMID: 33538338

[2] Thomas A Mohindroo C Giaccone G . Advancing therapeutics in small-cell lung cancer. Nat Cancer. (2025) 6:938–53. doi: 10.1038/s43018-025-00996-1, PMID: 40523990

[3] Riudavets M Cascetta P Planchard D . Targeting BRAF-mutant non-small cell lung cancer: Current status and future directions. Lung Cancer. (2022) 169:102–14. doi: 10.1016/j.lungcan.2022.05.014, PMID: 35696864

[4] Planchard D Besse B Peters S Felip E Bauer TT Jakopovic M . Dabrafenib plus trametinib in patients with previously untreated BRAF(V600E)-mutant metastatic non-small-cell lung cancer: an open-label, phase 2 trial. Lancet Oncol. (2017) 18:1307–16. doi: 10.1016/S1470-2045(17)30679-4, PMID: 28919011

[5] Reck M Remon J Hellmann MD . First-line immunotherapy for non-small-cell lung cancer. J Clin Oncol. (2022) 40:586–97. doi: 10.1200/JCO.21.01497, PMID: 34985920

[6] Kaur P Kaur R Dhanjal P Kumar V Siddiqui S Kaushik A . Promising combinatorial therapeutic strategies against non-small cell lung cancer. Cancers (Basel). (2024) 16. doi: 10.3390/cancers16122205, PMID: 38927911 PMC11201636

[7] Pavlidis N Pentheroudakis G . Cancer of unknown primary site. Lancet. (2012) 379:1428–35. doi: 10.1016/S0140-6736(11)61178-1, PMID: 22414598

[8] Hemminki K Hemminki A Försti A Sundquist J Sundquist K . Site-specific cancer deaths in cancer of unknown primary diagnosed with lymph node metastasis may reveal hidden primaries. Int J Cancer. (2013) 132:944–50. doi: 10.1002/ijc.27678, PMID: 22730111

[9] Fizazi K Greco FA Pavlidis N Pentheroudakis G . Cancers of unknown primary site: ESMO Clinical Practice Guidelines for diagnosis, treatment and follow-up. Ann Oncol. (2015) 26 Suppl 5:v133–8. doi: 10.1093/annonc/mdv305, PMID: 26314775

[10] Kamposioras K Pentheroudakis G Pavlidis NJE . Exploring the biology of cancer of unknown primary: breakthroughs and drawbacks. Eur J Clin Invest. (2013) 43:491–500. doi: 10.1111/eci.12062, PMID: 23480555

[11] Bochtler T Krämer AJF . Does cancer of unknown primary (CUP) truly exist as a distinct cancer entity?. Front Oncol. (2019) 9:402. doi: 10.3389/fonc.2019.00402, PMID: 31165045 PMC6534107

[12] Samir RM Osman GS El-Deftar MM . Diagnostic value of napsin A in small biopsy of non-small cell lung carcinoma. Egypt J Hosp Med. (2021) 83:1297–301. doi: 10.21608/ejhm.2021.165501

[13] Gou Q Gu R Wang J Zhou S . Novel therapeutic strategies for rare mutations in non-small cell lung cancer. Sci Rep. (2024) 14:10317. doi: 10.1038/s41598-024-61087-2, PMID: 38705930 PMC11070427

[14] J JS Boyle TA . Molecular pathology of lung cancer. Cold Spring Harb Perspect Med. (2022) 12. doi: 10.1101/cshperspect.a037812, PMID: 34751163 PMC8886739

[15] Harada G Cocco M Drilon A Oxnard GR . Rare molecular subtypes of lung cancer. Nat Rev Clin Oncol. (2023) 20:229–49. doi: 10.1038/s41571-023-00733-6, PMID: 36806787 PMC10413877

[16] Cardarella S Johnson JL Tsherniak A Collier K Kris MG Arcila ME . Clinical, pathologic, and biologic features associated with BRAF mutations in non–small cell lung cancer. Clin Cancer Res. (2013) 19:4532–40. doi: 10.1158/1078-0432.CCR-13-0657, PMID: 23833300 PMC3762878

[17] Poulikakos PI Sullivan RJ Yaeger R . Molecular pathways and mechanisms of BRAF in cancer therapy. Clin Cancer Res. (2022) 28:4618–28. doi: 10.1158/1078-0432.CCR-21-2138, PMID: 35486097 PMC9616966

[18] Young A Lyons J Miller AL Phan VT Rangel-Alarcón I McCormick F . Ras signaling and therapies. Adv Cancer Res. (2009) 102:1–17. doi: 10.1016/S0065-230X(09)02001-6, PMID: 19595305

[19] Thevakumaran N Lavoie H Critton DA Tebben A Marinier A Sicheri F . Crystal structure of a BRAF kinase domain monomer explains basis for allosteric regulation. Nat Struct Mol Biol. (2015) 22:37–43. doi: 10.1038/nsmb.2924, PMID: 25437913

[20] Chapman PB Hauschild A Robert C Haanen JB Ascierto PA Larkin J . Improved survival with vemurafenib in melanoma with BRAF V600E mutation. N Engl J Med. (2011) 364:2507–16. doi: 10.1056/NEJMoa1103782, PMID: 21639808 PMC3549296

[21] Villanueva J Vultur A Lee JT Somasundaram R Fukunaga-Kalabis M Radhakrishnan R . Acquired resistance to BRAF inhibitors mediated by a RAF kinase switch in melanoma can be overcome by cotargeting MEK and IGF-1R/PI3K. Cancer Cell. (2010) 18:683–95. doi: 10.1016/j.ccr.2010.11.023, PMID: 21156289 PMC3026446

[22] Planchard D Besse B Groen HJM Souquet P-J Quoix E Baik CS . Dabrafenib plus trametinib in patients with previously treated BRAFV600E-mutant metastatic non-small cell lung cancer: an open-label, multicentre phase 2 trial. Lancet Oncol. (2016) 17:984–93. doi: 10.1016/S1470-2045(16)30146-2, PMID: 27283860 PMC4993103

[23] Parisi C Planchard D . BRAF in non-small cell lung cancer: From molecular mechanisms to clinical practice. Cancer. (2025) 131 Suppl 1:e35781. doi: 10.1002/cncr.35781, PMID: 40172088

[24] Cohen JV Sullivan R.J.J.C.C.R . Developments in the space of new MAPK pathway inhibitors for BRAF-mutant melanoma. Clin Cancer Res. (2019) 25:5735–42. doi: 10.1158/1078-0432.CCR-18-0836, PMID: 30992297 PMC6774830

[25] Facchinetti F Lacroix L Mezquita L Scoazec J-Y Loriot Y Tselikas L . Molecular mechanisms of resistance to BRAF and MEK inhibitors in BRAFV600E non–small cell lung cancer. Eur J Cancer. (2020) 132:211–23. doi: 10.1016/j.ejca.2020.03.025, PMID: 32388065

[26] Hu-Lieskovan S Mok S Homet Moreno B Tsoi J Robert L Goedert L . Improved antitumor activity of immunotherapy with BRAF and MEK inhibitors in BRAF(V600E) melanoma. Sci Transl Med. (2015) 7:279ra41. doi: 10.1126/scitranslmed.aaa4691, PMID: 25787767 PMC4765379

[27] Ribas A Lawrence D Atkinson V Agarwal S Miller Jr WH Carlino MS . Combined BRAF and MEK inhibition with PD-1 blockade immunotherapy in BRAF-mutant melanoma. Nat Med. (2019) 25:936–40. doi: 10.1038/s41591-019-0476-5, PMID: 31171879 PMC8562134

[28] Dummer R Lebbé C Atkinson V Mandalà M Nathan PD Arance A . Combined PD-1, BRAF and MEK inhibition in advanced BRAF-mutant melanoma: safety run-in and biomarker cohorts of COMBI-i. Nat Med. (2020) 26:1557–63. doi: 10.1038/s41591-020-1082-2, PMID: 33020648

[29] Cheng S Zhang R Yang F Li J Zhao H Zhou L . Induction therapy with dabrafenib, trametinib, and PD-1 inhibitor and surgical conversion in unresectable stage III non-small cell lung cancers with BRAF V600E mutation: 2 cases. Lung Cancer. (2025) 205:108592. doi: 10.1016/j.lungcan.2025.108592, PMID: 40466464

