# Supplementary material for: Response to dabrafenib and trametinib combined with pembrolizumab in an elderly patient with lung adenocarcinoma of unknown primary harboring BRAF V600E mutation and high PD-L1 expression: a case report
Source: Front Immunol. 2025 Nov 17;16:1666461. doi: 10.3389/fimmu.2025.1666461 (PMC12665787; doi:10.3389/fimmu.2025.1666461)
Supplement: Supplementary file 1 [file DataSheet1.pdf]

CARE case report guidelines

CARE Checklist of information to include when writing a case report

CC BY-NC-ND

Topic	Item	Checklist item description	Reported on Line
Title	1	The diagnosis or intervention of primary focus followed by the words "case report"	1-4
Key Words	2	2 to 5 key words that identify diagnoses or interventions in this case report, including "case report"	23-24
Abstract (no references)	3a	Introduction: What is unique about this case and what does it add to the scientific literature?	27-24
	3b	Main symptoms and/or important clinical findings	49-52
	3c	The main diagnoses, therapeutic interventions, and outcomes	52-95
	3d	Conclusion—What is the main "take-away" lesson(s) from this case?	226-237
Introduction	4	One or two paragraphs summarizing why this case is unique (**may include references**)	97-141
Patient Information	5a	De-identified patient specific information	49-50
	5b	Primary concerns and symptoms of the patient	49-52
	5c	Medical, family, and psycho-social history including relevant genetic information	49-52
	5d	Relevant past interventions with outcomes	
Clinical Findings	6	Describe significant physical examination (PE) and important clinical findings	49-52
Timeline	7	Historical and current information from this episode of care organized as a timeline	84-95
Diagnostic Assessment	8a	Diagnostic testing (such as PE, laboratory testing, imaging, surveys).	58-83
	8b	Diagnostic challenges (such as access to testing, financial, or cultural)	63-67
	8c	Diagnosis (including other diagnoses considered)	74-75
	8d	Prognosis (such as staging in oncology) where applicable	91-95
Therapeutic Intervention	9a	Types of therapeutic intervention (such as pharmacologic, surgical, preventive, self-care)	87-89
	9b	Administration of therapeutic intervention (such as dosage, strength, duration)	87-89
	9c	Changes in therapeutic intervention (with rationale)	173-179
Follow-up and Outcomes	10a	Clinician and patient-assessed outcomes (if available)	91-95
	10b	Important follow-up diagnostic and other test results	91-95
	10c	Intervention adherence and tolerability (How was this assessed?)	94-95
	10d	Adverse and unanticipated events	94-95
Discussion	11a	A scientific discussion of the strengths AND limitations associated with this case report	97-224
	11b	Discussion of the relevant medical literature **with references**	97-224
	11c	The scientific rationale for any conclusions (including assessment of possible causes)	180-221
	11d	The primary "take-away" lessons of this case report (without references) in a one paragraph conclusion	226-237
Patient Perspective	12	The patient should share their perspective in one to two paragraphs on the treatment(s) they received	242-249
Informed Consent	13	Did the patient give informed consent? Please provide if requested	Yes ☑ No ☐
